# Targeted Protein Degradation Tools: Overview and Future Perspectives

**DOI:** 10.3390/biology9120421

**Published:** 2020-11-26

**Authors:** Yuri Prozzillo, Gaia Fattorini, Maria Virginia Santopietro, Luigi Suglia, Alessandra Ruggiero, Diego Ferreri, Giovanni Messina

**Affiliations:** 1 Department of Biology and Biotechnology “Charles Darwin”, Sapienza University of Rome, 00185 Rome, Italy; gaiafattorini18@gmail.com (G.F.); mariavirginia.santopietro@gmail.com (M.V.S.); luigisuglia888@gmail.com (L.S.); diegomattia.ferreri@gmail.com (D.F.); 2 Department of Clinical Infection, Microbiology and Immunology, Institute of Infection and Global Health, University of Liverpool, Liverpool L69 3BX, UK; alessandra.ruggiero@opbg.net; 3 Immune and Infectious Disease Division, Academic Department of Pediatrics (DPUO), Bambino Gesù Children’s Hospital, 00165 Rome, Italy; 4 Pasteur Institute of Italy, Fondazione Cenci-Bolognetti, 00161 Rome, Italy

**Keywords:** targeted protein inactivation (TPI), targeted protein degradation (TPD), dTAG, FKBP12, von Hippel–Lindau (VHL), degron, deGradFP, anchor-away, nanobody, nano-grad

## Abstract

**Simple Summary:**

Gene inactivation is a powerful strategy to study the function of specific proteins in the context of cellular physiology that can be applied for only non-essential genes since their DNA sequence is destroyed. On the other hand, perturbing the amount of the transcript can lead to incomplete protein depletion and generate potential off-target effects. Instead, targeting at the protein level is desirable to overcome these limitations. In the last decade, several approaches have been developed and wisely improved, including compartment delocalization tools and protein degradation systems. This review highlights the most recent advances in targeted protein inactivation (TPI) and focuses on a putative novel tool to specifically degrade endogenous genetically unmodified target protein.

**Abstract:**

Targeted protein inactivation (TPI) is an elegant approach to investigate protein function and its role in the cellular landscape, overcoming limitations of genetic perturbation strategies. These systems act in a reversible manner and reduce off-target effects exceeding the limitations of CRISPR/Cas9 and RNA interference, respectively. Several TPI have been developed and wisely improved, including compartment delocalization tools and protein degradation systems. However, unlike chemical tools such as PROTACs (PROteolysis TArgeting Chimeras), which work in a wild-type genomic background, TPI technologies require adding an aminoacidic signal sequence (tag) to the protein of interest (POI). On the other hand, the design and optimization of PROTACs are very laborious and time-consuming. In this review, we focus on anchor-away, deGradFP, auxin-inducible degron (AID) and dTAG technologies and discuss their recent applications and advances. Finally, we propose nano-grad, a novel nanobody-based protein degradation tool, which specifically proteolyzes endogenous tag-free target protein.

## 1. Introduction

Disruption of protein homeostasis is a powerful strategy for dissection specific protein functions in the context of cellular physiology. Two main approaches are currently used to suppress protein expression: gene knockout by CRISPR/Cas9 and gene knockdown by RNA interference (RNAi) [1].

While gene editing by CRISPR/Cas9 acts at the DNA level leading to a complete and irreversible depletion of the protein of interest (POI), RNAi induces reversible transcript degradation and, in turn, its reduction [2,3,4,5]. Moreover, both CRISPR/Cas9 and RNAi can generate off-target effects [6,7].

In the last two decades, RNAi has been widely used to achieve protein downregulation at the mRNA level when knockout approaches were not feasible for essential genes or a reversible experimental system was required. Nonetheless, a big disadvantage of this approach is that, when RNAi is induced, all protein products already translated remain unaffected. Overall, both CRISPR/Cas9 and RNAi strategies determine an indirect depletion of the POI and can trigger compensatory mechanisms [8]. Targeting at the protein level allows overcoming the limitations of gene inactivation and loss of function phenotypes with essential genes. The main advantages are it being acute and reversible so that the primary effects of protein depletion can be studied and distinguished from secondary effects or adaptive responses triggered by the slow silencing of gene expression [9]. Moreover, direct targeting of protein allows interfering specifically with its tridimensional conformations, including post-translational modifications and splice variant products. Additionally, this approach would allow the depletion of the POI in specific cellular compartments and modulate its concentration over time.

Therefore, protein degradation techniques arise from the need to control and analyze the function and involvement of gene products in a narrow time window of specific phases of various cellular activities, including cell division. The rapid effectiveness feasible by protein targeting can be exploited to downregulate POI in a stage-specific manner or when time is a relevant factor. In this context, cell division or Drosophila development studies perfectly match with the purpose of the protein interference. To obtain an accurate and efficient protein silencing, several strategies have been developed, such as protein displacement systems and targeted protein degradation (TPD) are emerging strategies which aim to achieve loss of function and proteolysis of POI, respectively. Protein displacement systems lead to inhibition of POI by compartment delocalization, while TPD exploits the powerful degradation signal peptide sequence (tag) to hijack POI to E3 ubiquitin ligases for ubiquitylation and consequentially proteasomal degradation by recruitment of the ubiquitin-proteasome system (UPS).

In this review, we focus on the widespread of various TPI systems and discuss their applications in understanding protein function. We report anchor-away (AA), an interesting displacement technology; deGradFP, auxin-inducible degron (AID) and dTAG degradation systems and their recent applications and advances. Finally, we propose a nanobody-based degrader (called nano-grad) to specifically degrade endogenous target protein, eliminating all the procedure steps for genetic manipulations.

## 2. Anchor-Away (AA)

Protein loss-of-function is a very important method to determine the role of a POI in different cellular and molecular mechanisms. Nuclear-localized proteins are involved in many important cellular processes such as DNA replication, DNA repair and transcription. Unlike all the other approaches described later, AA is not based on a degradation pathway and is mostly used to functionally inhibit nuclear POI. In this technique, a rapamycin-dependent treatment is used to sequester the POI away from its physiological cellular compartment to prevent its functionality. The system is reversible, and following the removal of the rapamycin-dependent treatment, the protein can return to its physiological location, resuming its functionality. The AA technique was first defined in *Saccharomyces cerevisiae* [10] and later applied in *Drosophila melanogaster* [11] and human cell lines [12]. In the latter application, mTOR inhibitor rapamycin is employed for its association with FK506 binding protein (FKBP12) and FKBP12-rapamycin-associated protein (FRAP) in the mammalian mTOR pathway, which controls cytokine-mediated cellular proliferation. Rapamycin bridges between the POI and the anchor protein, allowing functional inhibition of the target protein [13]. In detail, the FKBP12-rapamycin-binding-domain of FRAP (FRB) is fused to a specific POI, while the FKBP12 is fused to the anchor protein.

**Figure 1:**
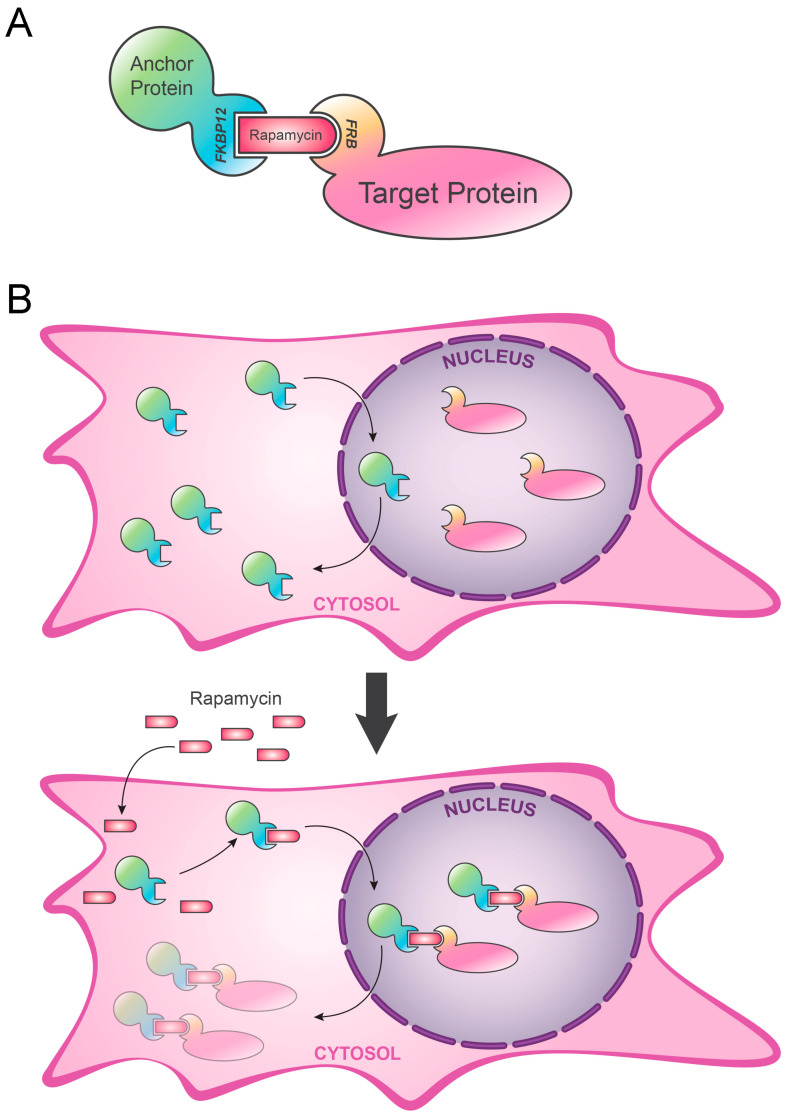
Anchor-away (AA). The mechanism of anchor-away is based on the functional inhibition of the target protein. (**A**) Target protein is fused with the FKBP12-rapamycin-binding-domain of FKBP12-rapamycin-associated protein (FRAP) (FRB), while the anchor protein is fused with FKBP12. (**B**) Added rapamycin acts as a bridge between FRB and FKBP12, and POI is functionally inhibited by its own displacement from the physiological localization. POI: protein of interest. Ub: ubiquitin.

One of the most prominent applications of the AA is to inhibit a nuclear-targeted protein by exporting it to the cytoplasm. In this case, the anchor protein must be an abundant cytoplasmic protein. A peculiar example is Rpl13A, a ribosomal protein that is physiologically imported into the nucleus, assembled to form the ribosome and then permanently exported to the cytoplasm. Thus, in the presence of rapamycin, the anchor protein (Rpl13A-FKBP12) interacts with the POI (fused with the C-terminal domain of FRAP), forming a stable ternary complex, which is exported in the cytoplasm where the POI protein is not able to properly work anymore (Figure 1).

Unfortunately, although AA is a very efficient strategy for the non-functional delocalization of nuclear proteins (towards cytoplasm), it is not always the case for the delocalization of cytoplasmic proteins towards the nucleus. Haruki et al. attempted to address this issue by proposing a histone-like anchor for delocalizing cytoplasmic proteins [10]. Changing course certainly represents a focal point in the future perspectives of this attractive technique.

## 3. deGradFP

**Figure 2:**
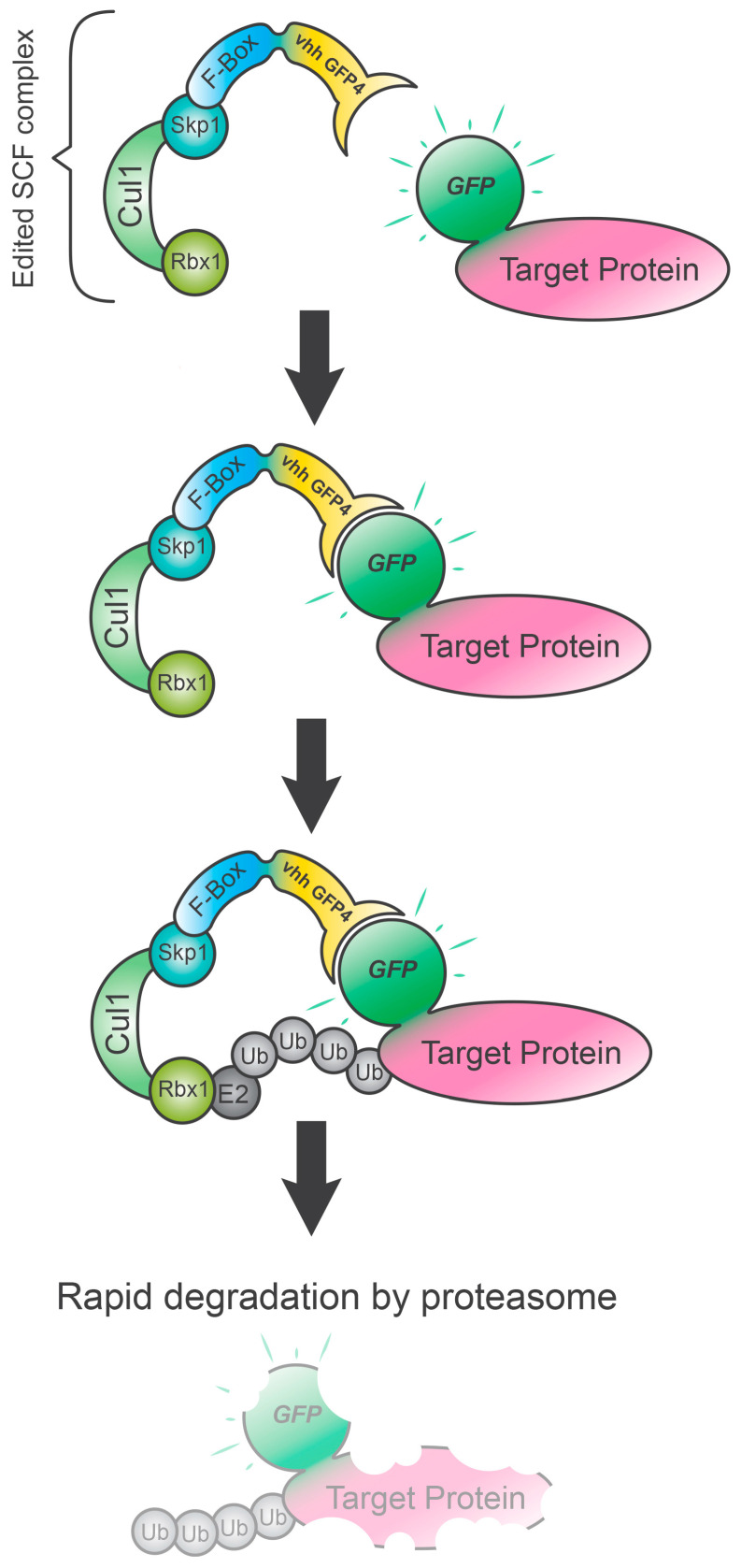
de(G)radation of fluorescent protein (deGradFP). The deGradFP technique relies on the specificity of the SCF complex given by the fused nanobody to a GFP-tag fused protein. The consequential poly-ubiquitination of the target protein results in a proteasome-dependent degradation. In this system, the SCF complex is composed of endogenous components: the adapter elements for the F-box protein (Skp1), cullin scaffold (Cul1) and the RING protein recruiting an E2 ligase (Rbx1). The specificity of the substrate is given by the FBP subunit target recognition (F-box), which is fused to the vhh-GFP4, an anti-GFP nanobody. Upon GFP-tagged protein recognition by F-box/vhh-GFP4, the E2 ubiquitin-protein ligase mediates the poly-ubiquitination of POI, which will be immediately degraded by the proteasome. POI: protein of interest. Ub: ubiquitin.

In the deGradFP technique, a transgenic adapter is fused to a specific nanobody (a natural single-domain antibody-containing only heavy chains from llama or alpaca), forming an E3 ubiquitin chimeric construct called the SCF (Skp, Cullin, F-box containing) complex. This recognizes a GFP-tag, which directs the polyubiquitination of the target GFP-protein which is then degraded by the proteasome pathway (Figure 2). The structure of the SCF complex normally is maintained in the DeGradFP except for the F-box protein subunit, which is substituted with an engineered inducible form (NSlmb) fused to a nanobody (VhhGFP4) against the fluorescent reporter proteins and some derivatives (Green Fluorescent Protein, GFP; Venus; Yellow Fluorescent Protein, YFP and Enhanced YFP, EYFP) [14,15]. The addition of a fluorescent protein allows fluorescence intensity to be measured to check POI downregulation in time-lapse experiments. Of note, any background noise can be excluded using a red fluorescent protein (RFP) tag signal, a negative control not recognized by the. Experimental evidence shows that the signal starts to decrease at ~30 min after induction, and only a residual 10% or less can be observed after ~3 h in most of the cases [14]. Moreover, Caussinus et al. found that the degradation efficiency is equivalent in different cellular compartments; therefore, nuclear, cytoplasmatic and transmembrane proteins (H2A.v::GFP, apterous ap::GFP, spaghetti squash sqh::GFP and crumb Crb::GFP), respectively, can be equally polyubiquitinated and targeted to the proteasome. This system is, however, not applicable in two cases. First, when the POI has a structure that could internalize the GFP tag, preventing the binding of the NSlmb-VhhGFP4 to the target. Second, the POI is part of a multiprotein complex that could hide the GFP tag, preventing accessibility by the SCF complex. In general, GFP is a big tag that could affect the functional folding of POI; therefore, preliminary rescue experiments are strongly recommended. It has also been observed that the GFP molecule itself is not recognized by this polyubiquitination complex unless associated with another protein or peptide (the smallest chain tested is the 3xNLS), suggesting a molecule recognition size limit for this system [14,15,16]. Since several transgenic stocks carrying GFP-tagged proteins for Drosophila and other model organisms already exist, deGradFP can be considered the most versatile system [14,17]. A more recent system based on the adaptor specificity of the E3-ubiquitin ligase has been proposed. In this case, the GFP nanobody VhhGFP4 is attached to SPOP E3 ubiquitin ligase that guides the degradation of nuclear protein more efficiently [18]. The evolutionary conservation of the complex indicated the possibility of implementing this SPOP-based deGradFP system in different organisms like *Drosophila melanogaster* [14], *Nicotiana tabacum* [19], *Danio rerio* [20], *Parhyale hawaiensis* [17] and animal cell lines [16].

## 4. Auxin-Inducible Degron System (AID)

Auxins such as the IAA (indol-3-acetic acid) are hormones that control many stages of growth and plant development. When auxins increase, it allows the transcription of the auxin-responsive genes through degradation of the transcriptional repressors AUX/IAA via the proteasome.

In this pathway, there are three essential components: the E3 ubiquitin ligase enzyme, the target proteins and the phytohormone. As explained above, the E3 ubiquitin ligase is the SCF (Skp1, Cullin 1 and F-box) complex. The F-box subunit can be modified according to different applications to mediate substrate specificity. TIR1 (transport inhibitor response 1) is a particular F-Box protein, which binds to auxins and leads to the target (AUX/IAA) recognition. Subsequently, an E2 ubiquitin-conjugating enzyme is recruited for polyubiquitination and degradation of the POI. The application of this degradation system in non-plant cells is based on the presence of SCF complex in all eukaryotes, but it is limited because of missing TIR1 orthologs.

**Figure 3:**
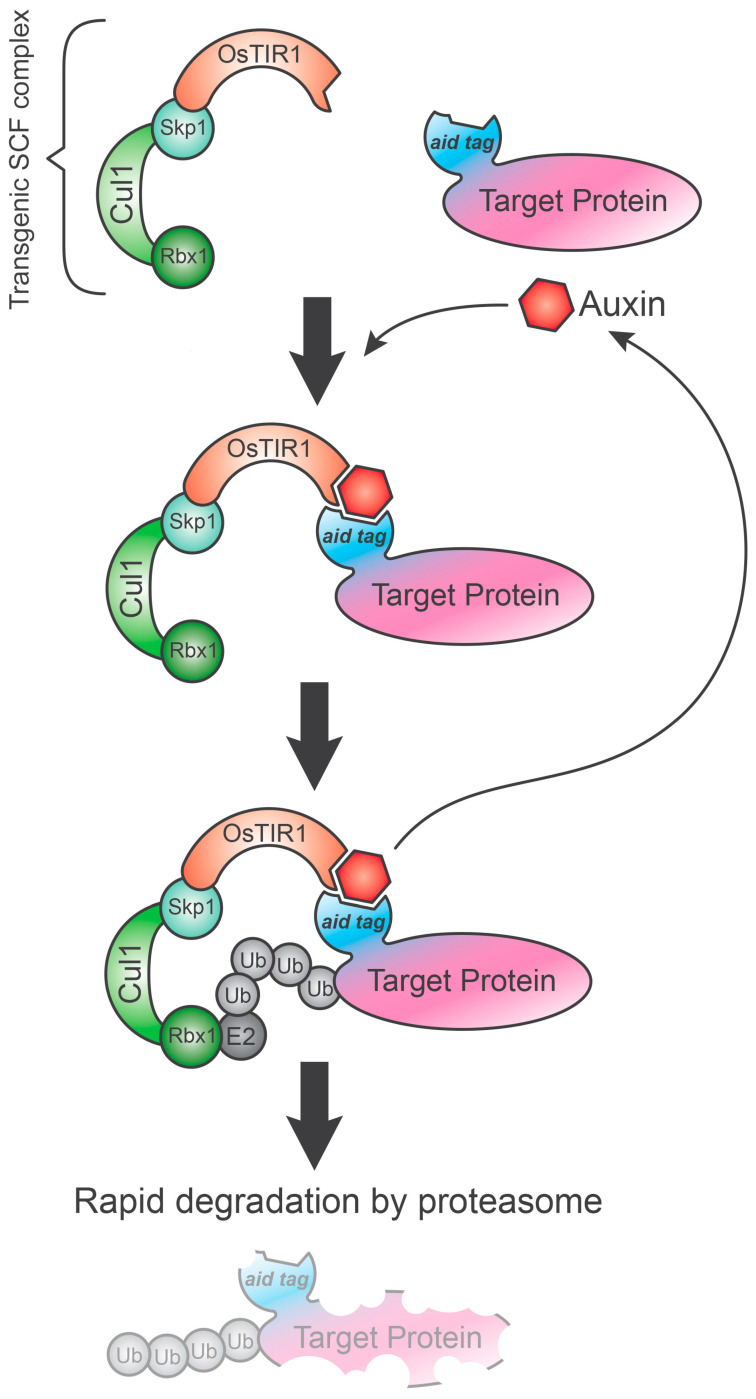
Auxin-inducible degradation (AID). The AID system requires the ectopic expression of both the OsTIR1 gene and aid-tagged protein of interest (POI). As shown in the figure, the exogenous OsTIR1 gene can form a functional transgenic SCF complex by connecting with the endogenous elements of the complex (Skp1, Cul1 and Rbx1). When auxin is added in the culture medium, it binds OsTIR1 triggering POI recognition and its subsequent polyubiquitination by an E2 ubiquitin-conjugating enzyme. The tagged protein is rapidly lead to the proteasome for degradation. POI: protein of interest. Ub: ubiquitin.

Using genetic manipulation, it was possible to mediate the ectopic expressions of both TIR1 and an AID tag sequence (derived from an AUX/IAA, recognized by TIR1) to tag an endogenous target protein. The exogenous TIR1 is able to form a functional SCF-TIR1 complex that can lead to the degradation of the tagged protein by the proteasome pathway in the presence of auxin [19] (Figure 3). This system was successfully used in many organisms, including yeast (*S. cerevisiae*), chicken (*G. gallus*), hamster (*M. auratus*) and human cells. In yeast, it has been demonstrated that the system works using the IAA17 (also known as AX3) peptide as an AID tag and the TIR1 adapter, both derived from *Arabidopsis thaliana* [21]. AtTIR1 denatured at temperature incubation required for animal cell culture [21]. Therefore, it was replaced with the *Oryza sativa* ortholog OsTIR1, which is more stable at high temperatures. When the auxin is supplied, the activation of the TIR1 protein induces a very rapid POI depletion, with a half-life of 10–20 min [22,23], which can be reverted by auxin removal [21].

However, the use of the natural auxin IAA is limited in some model organisms [24,25,26], and all of the secondary effects are abolished by using a synthetic auxin, the NAA (1-naphtalenicetic acid).

The AID system is extensively used to study the function of both non-essential and essential genes in *Saccharomyces cerevisiae*, *Drosophila melanogaster* and *Caenorhabditis elegans*, while its application in human cells has been limited because of complex AID-tagging of endogenous proteins, especially for essential proteins [21]. To overcome this problem, the CRISPR/Cas9-based genome editing tool has been exploited to fuse the AID tag to essential genes in human cell lines [27]. AID based degradation methods were used to differentiate between direct and indirect transcriptional targets of transcription factors [28]. Moreover, the introduction of an m-AID tag (minimum AID tag, 7-kDa) coupled to CRISPR/Cas9 approach has been successfully used in other studies that involved essential human genes with roles in cell division like APC4 [29] or Cdc7, a serine-threonine kinase involved in many processes including Aurora B activity stimulation [30], a master essential kinase required during mitosis. In some cases, to obtain a faster and more efficient POI degradation, the AID system has been coupled to Tet-OFF promoter system, in which the simultaneous IAA+doxycycline (dox) treatment results in a more rapid and complete POI degradation compared to a depletion mediated only by dox or IAA [31]. This combined system has been used in the investigation of essential human proteins like CDK2, a kinase essential for S-phase progression; cyclin A, partner of CDK1 and CDK2, involved in the control of the S-phase and mitosis; and TRIP13, a protein that regulates the spindle assembly checkpoint (SAC) [31].

A limitation of the original AID system is the premature degradation of the target when auxin is not added in the culture medium [32,33,34]. This auxin-independent degradation is a consequence of the high expression rate of TIR1. To overcome this problem, a Tet-Promoter in combination with the AID system was employed to put the Os-TIR1 gene under a tetracycline-regulated promoter [27]. However, the tet-OsTIR1 expression could be slow and have an influence on the degradation timing.

To prevent basal auxin-independent depletion of the target protein, many efforts were spent to develop an improved auxin-inducible degron system in which ARFs (auxin transcription factors) are co-expressed together with components of the original AID system. In plant cells, when the auxins level is low, ARFs bind AUX/IAA proteins [35]. Alternatively, when the auxins levels increase, TIR1 interacts with AUX/IAA (which are both lead to the proteasome), and ARFs proteins are released to regulate transcription of auxin-responsive genes. In this improvement, native levels of POI are preserved by the formation of a stable ARF-AID tag complex, which changes the conformation of the AID-tag, preventing its premature association with auxin-unbonded-TIR1 and POI degradation [36].

A more recent improvement of the original AID system is the generation of a bicistronic all-in-one plasmid that mediates the expression of TIR1 together with the AID tag fused POI in order to reduce genetic manipulation [37]. Although this new approach relies on random plasmid integration in the host genome, a POI expression comparable to that of the endogenous protein can be achieved. Moreover, the control of OsTIR1 and AID tagged POI expression by the same promoter determinates a balanced expression of them, avoiding that overexpressed TIR1 can lead to auxin-independent degradation.

## 5. Degradation TAG (dTAG) System

**Figure 4:**
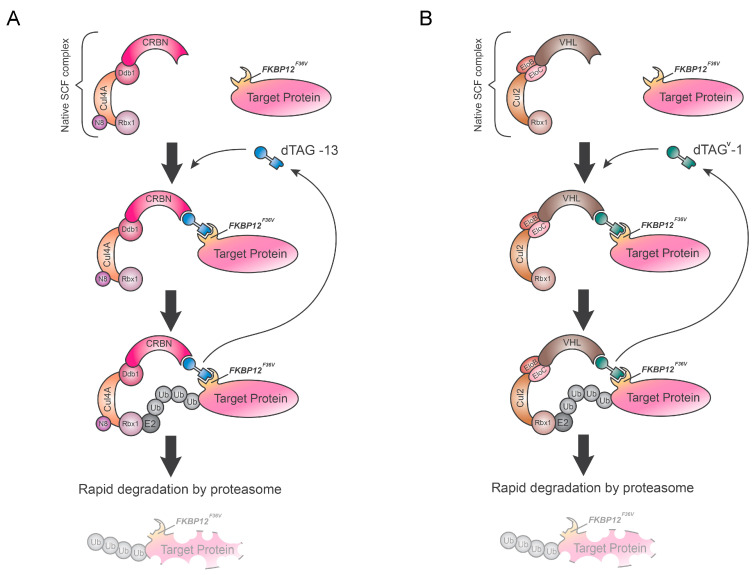
Degradation TAG (dTAG). (**A**) Heterobifunctional dTAG-13 molecules engage FKBP12^F36V^-fused POI and cereblon (CRBN), hijacking it towards endogenous proteasome machinery for POI degradation. dTAG-13 contains AP1867 and thalidomide, FKBP12^F36V^ and CRBN selective ligands, respectively. CRL4–CRBN E3 ubiquitin ligase recruited by dTAG-13 is composed of cullin scaffold (CUL4A), adaptor protein (DDB1), substrate receptor (CRBN), the RING protein (Rbx1) recruiting an E2 ligase and N8 ubiquitin-like protein (NEDD8). (**B**) dTAG^V^-1 molecule recruits the von Hippel–Lindau (VHL) E3 ligase complex, increasing dTAG system efficiency. VHL complex consists of a cullin scaffold (Cul2), two adaptor proteins (EloB and EloC), a substrate receptor (VHL), and Rbx1. POI: protein of interest. Ub: ubiquitin.

The degradation TAG system is a technology for rapid, reversible and selective depletion of a POI developed by Nabet et al. [38]. This approach leverages a modified version of the FKBP12 tag (FKBP12^F36V^) used in the anchor-away system and needs three major components: an FKBP12^F36V^-fused POI, a small synthetic molecule, a defined degrader, and the endogenous E3 ligase complex (Figure 4). The target protein is fused with the 12-kDa cytosolic prolyl isomerase engineered variant (FKBP12^F36V^) through transgene expression or CRISPR-mediated locus-specific knock-in [39,40]. A heterobifunctional degrader (e.g., dTAG-13) recruits FKBP12^F36V^-fused POI to cereblon (CRBN), the recognition unit of CRL4-CRBN E3 ubiquitin ligase complex, leading to exclusive POI degradation by the proteasome. In brief, researchers have developed a series of degrader molecules consisting of AP1867, a synthetic FKBP12^F36V^ selective ligand, and thalidomide binding CRBN, connected by different linkers.

The dTAG system was first employed to evaluate the consequences of acute degradation of ENL and MELK, a transcriptional regulator and a promoting proliferation kinase, respectively [41,42]; and later it was successfully tested in cells to rapidly and selectively degrade a panel of FKBP12^F36V^-fused chimeras like BRD4, HDAC1, EZH2, MYC, PLK1, and KRASG12V. Reported data shows specific variation rates of POI degradation depending on target subcellular compartmentalization. Moreover, Nabet et al. extended the dTAG-strategy to in vivo targeted degradation studies using mouse models. Recent applications of this technology were adapted to rapidly deplete all IE2 protein isoforms, to elucidate the role of these proteins in HCMV late infection [43], and also to degrade solute carrier (SLC) proteins, the largest class of transporters with multipass transmembrane domain topology [44]. Based on reported data, the dTAG platform is a versatile system triggering selective depletion without off-target effects [45]. This powerful approach would be an ideal validation strategy of therapeutic targets in in vivo applications [38,41].

More recently, Nabet et al. [46] have expanded the suite of dTAG molecules by developing dTAG^V^-1, a degrader that engages the von Hippel–Lindau (VHL) E3 ligase complex. This second generation of dTAG overcomes limitations of dTAG-13 in the degradation of several proteins, exemplified by EWS/FLI, a driver of Ewing sarcoma. dTAG^V^-1 compared to dTAG-13 show an increased pharmacokinetic and pharmacodynamic profile with a longer half-life, a greater exposure, and an improved duration of degradation. This implemented tool makes the dTAG strategy universally applicable and a good candidate for the most efficient and least time-consuming TPD approach.

## 6. A Future Perspective: The Nano-Grad System

During the last decade, the field of TPI has had massive growth and impact rapidly in cell and developmental biology. We discussed different approaches to modulate protein functionality and study their physiological roles. Although the anchor-away system has the advantage of being an inducible/reversible system applicable to isolate the specific role of a target protein with multiple functions in different cell compartments (cytoplasm and nucleus) [47], it also has some disadvantages because it can only be applied to nuclear proteins. Thus, more versatile methods have been developed. One possible alternative is based on the use of the ubiquitination pathway to induce targeted protein degradation via the proteasome. Caussinus et al. [14] engineered an anti-GFP nanobody fused to F-box (deGradFP) to channel GFP-tagged protein to the proteasome for degradation. This strategy proved to be highly beneficial for time-lapse microscopy to follow POI degradation in vivo. Although deGradFP is more suitable for any protein, it is not a reversible system and requires the addition of a relatively big tag (GFP) to POI, risking functional folding. To reduce tag size and genetic modifications, the Auxin-inducible degron and dTAG systems were developed.

While the AID is an auxin-dependent degradation system which still requires expression of an adaptor from planta (TIR1), the dTAG does not need any added transgenic element because it exploits native degradation pathway triggered by a very permeable and small heterobifunctional degrader (e.g., dTAG-13) approximating tagged-POI and proteasome machinery. Even though dTAG represents the latest frontier in TPD, it still requires the POI to be fused to a tag. Therefore, cloning and stable cell line generation procedures based on knock-in CRISPR/Cas9 tools cannot be avoided. Additionally, any tag size can potentially affect the functionality of the POI. In our experience, both 3xFlag (≃3 × 1-kDa) and GFP (≃27-KDa) fusions of *Drosophila* Yeti protein fail to rescue fly viability, indicating that Flag or GFP tag compromise its own physiological function [48]. Hence the need to eliminate the protein tagging process and flexibility of the system.

Addressing all these limitations, Bery et al. set up an original cell-based screen to identify nanobodies that selectively degrade the RHOB-GTP fraction by engineering them with a functionalized F-box domain [49]. The F-box/intrabody-mediated protein degradation represents the first approach to selectively target tag-free POI, an active form of small GTPases or other proteins with multiple cellular activities, although the preliminary nanobodies identification process is difficult and highly time-consuming [49]. A further attempt was made with TRIM away and proteolysis-targeting chimeras (PROTACs) systems.

In TRIM away, POI-specific antibody brings between TRIM21 and POI together to mark POI for degradation through ubiquitination. TRIM21 is an E3 ubiquitin ligase that binds with high affinity to the Fc domain of antibodies. This approach is extremely versatile but not feasible for large-scale use as the antibody must be internalized by injection or electroporation [50]. Unlike most of the degradation systems, PROTACs do not necessitate genetic modification of target gene as they work against endogenous POIs by using small bivalent molecules that are able to simultaneously bind POI and an E3 ligase component, ultimately leading to POI ubiquitination and degradation. Undoubtedly, the identification or development of PROTACs that can freely pass the cell membrane and target different E3-ligase and POI requires great effort, and it is an extremely high time-consuming procedure [51].

**Figure 5:**
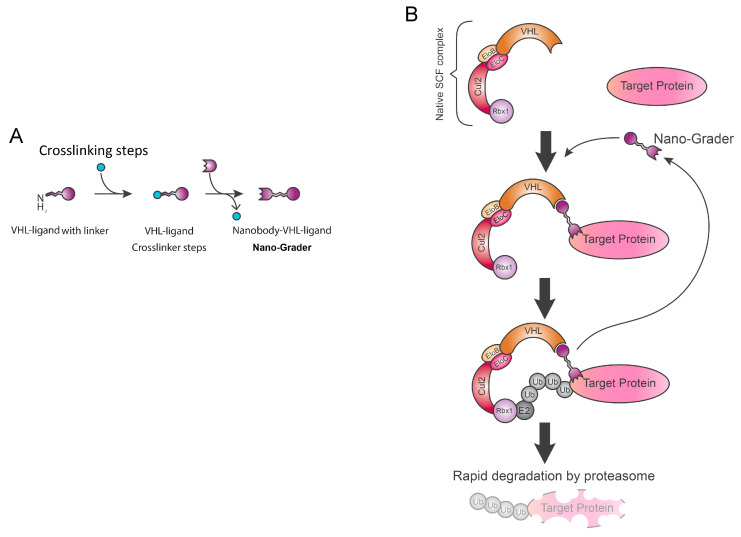
Nano-grad (NG). *(***A**) Nano-grad contains VHL selective ligand, an anti-POI specific nanobody and arginine-rich tails for cellular permeabilization. *(***B**) Heterobifunctional nano-grader molecules bring together the von Hippel–Lindau (VHL) E3 ligase complex and POI catalyzing proteasomal degradation of the target protein.

In order to overcome the above-mentioned limitations, we are working on the optimization of an innovative strategy exploiting the efficiency of nanobody and dTAG system to approach close together POI and proteasome machinery to stimulate rapid degradation: nano-grad [52]. In our model (Figure 5), the VHL ligand is covalently linked to POI-specific VHH (nanobody) to generate a new type of degrader, which we call nano-grader (NG) (Figure 5A). Likewise, dTAG^V^-1, the hetero-bifunctional NG, is able to approximate the VHL subunit to free-tag POI and trigger its rapid degradation via the proteasome. A crucial step for the success of this method is the internalization of the NG into the cells. To allow this passage, we propose the generation of cell-permeable nanobodies by site-specific attachment of a cyclic arginine-rich cell-penetrating peptide, as previously done by Here et al. [53]. Theoretically, the nano-grader strategy may represent a highly versatile system, as nanobodies against every POI could accept these modifications to develop themselves into NG and trigger tag-free target protein degradation.

## 7. Conclusions

Although the nano-grad innovative approach has several disadvantages, which are outweighed by the many advantages (Table 1), one limit could be the strength of the bond between antigen and nanobody, which can vary quite substantially. Another aspect is that needs to be discussed is that proteasome machinery could potentially incorporate NG for degradation, leading to a progressive accumulation of VHL ligand molecules, which result in toxicity for the cell, such as the unidirectional interaction with the only proteasome, which makes it inactive in a dominant-negative manner. On the other hand, cell machinery could compensate for this toxic effect by physiologically eliminating the thalidomide. Nonetheless, the nano-grad approach contains elements of novelty and advancement, which are worth to be tested and optimized. In conclusion, nano-grad could become the last generation of TPI and open the way towards forsaking the need to tag the POI and get tangled up with cumbersome genetic manipulations.

## Figures and Tables

**Table 1:** Advantages and disadvantages of targeted protein silencing systems.

System	Tag Size	Degrader (MW g/mol)	Organisms	Advantages	Disadvantages	Reversible/Inducible	Transgenic Elements
Anchor-away	12-kDa	Rapamycin (914)	- Fly - Yeast - Human cell lines	- Functional inhibition of multiple tagged proteins - No stress response mechanism is activated - In vivo applicability - High selectivity	- Usable only for nuclear POIs - Requires engineering fusion between proteins - Several pilot experiments are needed to understand the concentration of rapamycin - Long-time degradation (the silencing is detectable 6 h after treatment)	Yes/Yes	2
deGradFP	27-kDa	-	- Zebrafish - Crustacean - Fly - Plants - Animal cell lines	- Nuclear, cytoplasmatic and trans-membrane targets available - In vivo applicability - Possibility to follow the degradation process by fluorescence - Availability of large libraries of GFP::proteins for different model organisms	- Long-time degradation (less the 10% of the EGFP signal after 3 h) - Requires genetic engineering (endogenous expression of GFP::POI); - tag size - Some fusion proteins cannot be detected by the system	No/No	2
AID system	7-kDa	IAA (175) NAA (186)	- Fly - Worm - Yeast - Chicken, Human, and murine cell lines	- Rapid POI degradation - Useful for both nuclear and cytoplasmatic proteins - Two kind of inducers (natural or synthetic auxins) - Couplable with other systems (CRISPR-Cas, Tet promoters) - Preserves native levels of POI	- Usable only in non-plant cells - Limited by the presence of only a few orthologs of TIR1 - Requires genetic manipulation (TIR1 and tag-fused protein expression)	Yes/Yes	2
dTAG system	12-kDa	dTAG-13 (1049) dTAG^V^-1 (1361)	- Mouse - Human and murine cell lines	- High selectivity - Rapid POI degradation - Small tag size - In vivo applicability - Excellent pharmacokinetic and pharmacodynamic properties (long half-life of degrader and great exposure)	- Different rates of POI degradation depending on subcellular compartments - Limited to the cell systems in which CRISPR/Cas9 modifications are feasible - Tested in a few organisms	Yes/Yes	1
nano-grad	No	N/A	N/A	- Preserves native levels of POI - In vivo applicability * - No genetic manipulation is required	- Nano-grader may also be degraded by proteasome *	Yes/Yes	0

* These represent only putative advantages/disadvantages since they have not yet been experimentally verified. N/A = Not Available.

## References

[1] Fire A., Xu S., Montgomery M.K., Kostas S.A., Driver S.E., Mello C.C. (1998). Potent and specific genetic interference by double-stranded RNA in *Caenorhabditis elegans*. Nature.

[2] Prozzillo Y., Delle Monache F., Ferreri D., Cuticone S., Dimitri P., Messina G. (2019). The True Story of Yeti, the “Abominable” Heterochromatic Gene of *Drosophila melanogaster*. Front. Physiol..

[3] Messina G., Atterrato M.T., Fanti L., Giordano E., Dimitri P. (2016). Expression of human Cfdp1 gene in Drosophila reveals new insights into the function of the evolutionarily conserved BCNT protein family. Sci. Rep..

[4] Messina G., Atterrato M.T., Prozzillo Y., Piacentini L., Losada A., Dimitri P. (2017). The human Cranio Facial Development Protein 1 (Cfdp1) gene encodes a protein required for the maintenance of higher-order chromatin organization. Sci. Rep..

[5] Taylor E., Alqadri N., Dodgson L., Mason D., Lyulcheva E., Messina G., Bennett D. (2017). MRL proteins cooperate with activated Ras in glia to drive distinct oncogenic outcomes. Oncogene.

[6] Jackson A.L., Bartz S.R., Schelter J., Kobayashi S.V., Burchard J., Mao M., Li B., Cavet G., Linsley P.S. (2003). Expression profiling reveals off-target gene regulation by RNAi. Nat. Biotechnol..

[7] Wang Y., Wang M., Zheng T., Hou Y., Zhang P., Tang T., Wei J., Du Q. (2020). Specificity profiling of CRISPR system reveals greatly enhanced off-target gene editing. Sci. Rep..

[8] Rossi A., Kontarakis Z., Gerri C., Nolte H., Holper S., Kruger M., Stainier D.Y. (2015). Genetic compensation induced by deleterious mutations but not gene knockdowns. Nature.

[9] Roth S., Fulcher L.J., Sapkota G.P. (2019). Advances in targeted degradation of endogenous proteins. Cell. Mol. Life Sci..

[10] Haruki H., Nishikawa J., Laemmli U.K. (2008). The anchor-away technique: Rapid, conditional establishment of yeast mutant phenotypes. Mol. Cell.

[11] Bosch P.S., Pepperl J., Basler K. (2020). Anchor Away—A Fast, Reliable and Reversible Technique to Inhibit Proteins in *Drosophila melanogaster*. G3.

[12] Samwer M., Schneider M.W.G., Hoefler R., Schmalhorst P.S., Jude J.G., Zuber J., Gerlich D.W. (2017). DNA Cross-Bridging Shapes a Single Nucleus from a Set of Mitotic Chromosomes. Cell.

[13] Chen J., Zheng X.F., Brown E.J., Schreiber S.L. (1995). Identification of an 11-kDa FKBP12-rapamycin-binding domain within the 289-kDa FKBP12-rapamycin-associated protein and characterization of a critical serine residue. Proc. Natl. Acad. Sci. USA.

[14] Caussinus E., Kanca O., Affolter M. (2011). Fluorescent fusion protein knockout mediated by anti-GFP nanobody. Nat. Struct. Mol. Biol..

[15] Caussinus E., Affolter M. (2016). deGradFP: A System to Knockdown GFP-Tagged Proteins. Methods Mol. Biol..

[16] Caussinus E., Kanca O., Affolter M. (2013). Protein knockouts in living eukaryotes using deGradFP and green fluorescent protein fusion targets. Curr. Protoc. Protein Sci..

[17] Fedorova S.A., Dorogova N.V. (2020). Protein trap: A new Swiss army knife for geneticists?. Mol. Biol. Rep..

[18] Shin Y.J., Park S.K., Jung Y.J., Kim Y.N., Kim K.S., Park O.K., Kwon S.H., Jeon S.H., Trinh le A., Fraser S.E. (2015). Nanobody-targeted E3-ubiquitin ligase complex degrades nuclear proteins. Sci. Rep..

[19] Baudisch B., Pfort I., Sorge E., Conrad U. (2018). Nanobody-Directed Specific Degradation of Proteins by the 26S-Proteasome in Plants. Front. Plant Sci..

[20] Yamaguchi N., Colak-Champollion T., Knaut H. (2019). zGrad is a nanobody-based degron system that inactivates proteins in zebrafish. Elife.

[21] Nishimura K., Fukagawa T., Takisawa H., Kakimoto T., Kanemaki M. (2009). An auxin-based degron system for the rapid depletion of proteins in nonplant cells. Nat. Methods.

[22] Holland A.J., Fachinetti D., Han J.S., Cleveland D.W. (2012). Inducible, reversible system for the rapid and complete degradation of proteins in mammalian cells. Proc. Natl. Acad. Sci. USA.

[23] Lambrus B.G., Uetake Y., Clutario K.M., Daggubati V., Snyder M., Sluder G., Holland A.J. (2015). p53 protects against genome instability following centriole duplication failure. J. Cell Biol..

[24] Kwon J.Y., Hong M., Choi M.S., Kang S., Duke K., Kim S., Lee S., Lee J. (2004). Ethanol-response genes and their regulation analyzed by a microarray and comparative genomic approach in the nematode Caenorhabditis elegans. Genomics.

[25] Papagiannakis A., de Jonge J.J., Zhang Z., Heinemann M. (2017). Quantitative characterization of the auxin-inducible degron: A guide for dynamic protein depletion in single yeast cells. Sci. Rep..

[26] Camlin N.J., Evans J.P. (2019). Auxin-inducible protein degradation as a novel approach for protein depletion and reverse genetic discoveries in mammalian oocytesdagger. Biol. Reprod..

[27] Natsume T., Kiyomitsu T., Saga Y., Kanemaki M.T. (2016). Rapid Protein Depletion in Human Cells by Auxin-Inducible Degron Tagging with Short Homology Donors. Cell Rep..

[28] Muhar M., Ebert A., Neumann T., Umkehrer C., Jude J., Wieshofer C., Rescheneder P., Lipp J.J., Herzog V.A., Reichholf B. (2018). SLAM-seq defines direct gene-regulatory functions of the BRD4-MYC axis. Science.

[29] Lok T.M., Wang Y., Xu W.K., Xie S., Ma H.T., Poon R.Y.C. (2020). Mitotic slippage is determined by p31(comet) and the weakening of the spindle-assembly checkpoint. Oncogene.

[30] Ito S., Goto H., Kuniyasu K., Shindo M., Yamada M., Tanaka K., Toh G.T., Sawa M., Inagaki M., Bartek J. (2019). Cdc7 kinase stimulates Aurora B kinase in M-phase. Sci. Rep..

[31] Ng L.Y., Ma H.T., Liu J.C.Y., Huang X., Lee N., Poon R.Y.C. (2019). Conditional gene inactivation by combining tetracycline-mediated transcriptional repression and auxin-inducible degron-mediated degradation. Cell Cycle.

[32] Morawska M., Ulrich H.D. (2013). An expanded tool kit for the auxin-inducible degron system in budding yeast. Yeast.

[33] Nishimura K., Fukagawa T. (2017). An efficient method to generate conditional knockout cell lines for essential genes by combination of auxin-inducible degron tag and CRISPR/Cas9. Chromosome Res. Int. J. Mol. Supramol. Evol. Asp. Chromosome Biol..

[34] Zasadzinska E., Huang J., Bailey A.O., Guo L.Y., Lee N.S., Srivastava S., Wong K.A., French B.T., Black B.E., Foltz D.R. (2018). Inheritance of CENP-A Nucleosomes during DNA Replication Requires HJURP. Dev. Cell.

[35] Guilfoyle T.J., Hagen G. (2007). Auxin response factors. Curr. Opin. Plant Biol..

[36] Sathyan K.M., McKenna B.D., Anderson W.D., Duarte F.M., Core L., Guertin M.J. (2019). An improved auxin-inducible degron system preserves native protein levels and enables rapid and specific protein depletion. Genes Dev..

[37] Yesbolatova A., Saito Y., Kanemaki M.T. (2020). Constructing Auxin-Inducible Degron Mutants Using an All-in-One Vector. Pharmaceuticals.

[38] Nabet B., Roberts J.M., Buckley D.L., Paulk J., Dastjerdi S., Yang A., Leggett A.L., Erb M.A., Lawlor M.A., Souza A. (2018). The dTAG system for immediate and target-specific protein degradation. Nat. Chem. Biol..

[39] Clackson T., Yang W., Rozamus L.W., Hatada M., Amara J.F., Rollins C.T., Stevenson L.F., Magari S.R., Wood S.A., Courage N.L. (1998). Redesigning an FKBP-ligand interface to generate chemical dimerizers with novel specificity. Proc. Natl. Acad. Sci. USA.

[40] Brand M., Winter G.E. (2019). Locus-Specific Knock-In of a Degradable Tag for Target Validation Studies. Methods Mol. Biol..

[41] Erb M.A., Scott T.G., Li B.E., Xie H., Paulk J., Seo H.S., Souza A., Roberts J.M., Dastjerdi S., Buckley D.L. (2017). Transcription control by the ENL YEATS domain in acute leukaemia. Nature.

[42] Huang H.T., Seo H.S., Zhang T., Wang Y., Jiang B., Li Q., Buckley D.L., Nabet B., Roberts J.M., Paulk J. (2017). MELK is not necessary for the proliferation of basal-like breast cancer cells. Elife.

[43] Li M., Ball C.B., Collins G., Hu Q., Luse D.S., Price D.H., Meier J.L. (2020). Human cytomegalovirus IE2 drives transcription initiation from a select subset of late infection viral promoters by host RNA polymerase II. PLoS Pathog..

[44] Bensimon A., Pizzagalli M.D., Kartnig F., Dvorak V., Essletzbichler P., Winter G.E., Superti-Furga G. (2020). Targeted Degradation of SLC Transporters Reveals Amenability of Multi-Pass Transmembrane Proteins to Ligand-Induced Proteolysis. Cell Chem. Biol..

[45] Mayor-Ruiz C., Winter G.E. (2019). Identification and characterization of cancer vulnerabilities via targeted protein degradation. Drug discovery today. Technologies.

[46] Nabet B., Ferguson F.M., Seong B.K.A., Kuljanin M., Leggett A.L., Mohardt M.L., Robichaud A., Conway A.S., Buckley D.L., Mancias J.D. (2020). Rapid and direct control of target protein levels with VHL-recruiting dTAG molecules. Nat. Commun..

[47] Messina G., Prozzillo Y., Monache F.D., Santopietro M.V., Atterrato M.T., Dimitri P. (2020). ATPase SRCAP is a new player in cell division, uncovering molecular aspects of Floating-Harbor syndrome. BioRxiv.

[48] Messina G., Damia E., Fanti L., Atterrato M.T., Celauro E., Mariotti F.R., Accardo M.C., Walther M., Verni F., Picchioni D. (2014). Yeti, an essential *Drosophila melanogaster* gene, encodes a protein required for chromatin organization. J. Cell Sci..

[49] Bery N., Keller L., Soulie M., Gence R., Iscache A.L., Cherier J., Cabantous S., Sordet O., Lajoie-Mazenc I., Pedelacq J.D. (2019). A Targeted Protein Degradation Cell-Based Screening for Nanobodies Selective toward the Cellular RHOB GTP-Bound Conformation. Cell Chem. Biol..

[50] Clift D., McEwan W.A., Labzin L.I., Konieczny V., Mogessie B., James L.C., Schuh M. (2017). A Method for the Acute and Rapid Degradation of Endogenous Proteins. Cell.

[51] Sakamoto K.M., Kim K.B., Kumagai A., Mercurio F., Crews C.M., Deshaies R.J. (2001). Protacs: Chimeric molecules that target proteins to the Skp1-Cullin-F box complex for ubiquitination and degradation. Proc. Natl. Acad. Sci. USA.

[52] Prozzillo Y., Cingolani A., Messina G. (2020). Department of Biology and Biotechnology “Charles Darwin”.

[53] Herce H.D., Schumacher D., Schneider A.F.L., Ludwig A.K., Mann F.A., Fillies M., Kasper M.A., Reinke S., Krause E., Leonhardt H. (2017). Cell-permeable nanobodies for targeted immunolabelling and antigen manipulation in living cells. Nat. Chem..

